# Effects of agro-forestry systems on the physical and chemical characteristics of green coffee beans

**DOI:** 10.3389/fnut.2023.1198802

**Published:** 2023-07-12

**Authors:** Su Xu, Yuze Liu, Zhenchun Sun, Guangjing Chen, Fengwei Ma, Ni Yang, Elias de Melo Virginio Filho, Ian D. Fisk

**Affiliations:** ^1^Food and Pharmaceutical Engineering Institute, Guiyang University, Guiyang, Guizhou, China; ^2^Division of Food Sciences, University of Nottingham, Sutton Bonington Campus, Loughborough, Leicestershire, United Kingdom; ^3^Centro Agronómico Tropical de Investigación y Enseñanza, CATIE, Turrialba, Costa Rica

**Keywords:** agriculture, shade trees, organic management practice, coffee, physicochemical properties

## Abstract

Twenty agroforestry systems consisting of different management practices (conventional and organic) and shade types were set up for coffee plantations in 2,000 at the Tropical Agricultural Research and Higher Education Center (CATIE), Turrialba, Costa Rica. The physical (density, bulk density, moisture content, and roasting loss) and chemical attributes (mineral, total lipid, fatty acids, caffeine, and carbohydrate contents) of harvested green coffee beans were investigated. The full sun and *Erythrina* shade tree systems significantly improved (*p* < 0.05) the density of the green coffee beans and decreased (*p* < 0.05) the moisture content and roasting loss of the green coffee beans. The intensive organic (IO) management practice significantly increased some mineral contents, such as K, P, and Ca, in green coffee beans. The full sun system also significantly promoted (*p* < 0.05) some mineral contents, such as Ca and Mn, in green coffee beans. In terms of total lipid and fatty acids (FAs), compared with the moderate conventional (MC) management practice, the IO management practice was beneficial as it significantly increased (*p* < 0.05) the total lipid and FAs contents in the green coffee beans, while the *Erythrina* shade tree system significantly increased (*p* < 0.05) the total lipid and FAs contents of green coffee beans more efficiently than the other shade types. The caffeine content of green coffee beans was significantly higher (*p* < 0.05) under the intensive conventional (IC) and IO management practices than under the MC management practice and higher under the full sun system than under the shaded system. The *Erythrina* shade tree system significantly improved (*p* < 0.05) the carbohydrate content of green coffee beans. Overall, in consideration of sustainability, the IO management practice associated with the *Erythrina* shade tree system would be a useful combination for the local farmers to grow coffee trees.

## 1. Introduction

Coffee is a major export product for developing countries and provides livelihoods for millions of people, constituting a major income for many countries in South and Central America, Africa, and Southeast Asia (1). It has been reported that the physical and chemical characteristics of green coffee beans are strongly correlated with environmental conditions (e.g., altitude, humidity, and sunlight), shade cover percentages, and agricultural practices (2–4). Nonetheless, field management methods are still highly conflicted between the physicochemical and organoleptic properties of coffee and shades and agricultural practices.

Somporn et al. (2) suggested that the weight and size of green coffee beans increased significantly under shade trees than under the full sun. Furthermore, lychee shade improved green coffee bean yield, total phenolic content, and antioxidant activity, while the full sun system promoted the generation of vanillic and caffeic acids. Similarly, Worku et al. (3) investigated the effects of altitude, shade, and postharvest processing on the content of biochemical substances and the quality of green coffee beans. It has been indicated that there are complicated interaction effects among altitude, shade levels, and postharvest processing on the biochemical composition of green coffee beans. For example, sucrose content increased significantly with altitude for wet-processed green coffee beans, but not for dry-processed green coffee beans; the sucrose content of green coffee beans grown without shade increased significantly with altitude. In contrast, Cassamo et al. (4) found that the physicochemical properties of green coffee beans were scarcely affected by altitude and light conditions (shade or full sun).

Management practices, especially organic and conventional fertilizers, have been proven to affect the yield and quality of green coffee beans (5–7). An increase in productivity has been achieved by using a large number of external agrochemical inputs and shortcutting ecological cycles (5, 8), which contribute to environmental pollution, degradation of soils (1, 6), and health hazards (7). The high annual production cost caused by the extensive use of these external inputs cannot be reduced easily, resulting in the vulnerability of coffee farmers to the unstable international coffee market (5). Nevertheless, environment, health, and demand for sustainable agricultural products such as organic coffee have led to 30% of coffee production being sustainably certified (9). Moreover, organic coffee production and certification have reduced the vulnerability of the income of coffee farmers to seasonal changes in coffee price and raw material costs (10), especially as farmers experimented with organic and shaded coffee production methods (5, 10). However, it is necessary to explore the interactions between management practices (organic and conventional fertilizers) and the physicochemical properties of green coffee beans.

Therefore, the aim of this research was to investigate the relationships between the agroforestry systems, including shade and fertilizers, and the physicochemical properties of green coffee beans, and attempt to explain whether the organic agroforestry system is more suitable for the production of green coffee beans.

## 2. Materials and methods

### 2.1. Agroforestry methodologies

This study was conducted at the Tropical Agricultural Research and Higher Education Center (CATIE), Turrialba, Costa Rica (9°53′44” N, 83°40′7” W, CATIE, Turrialba, Costa Rica) in 2000. According to the definition, it has a low elevation (600 m above sea level) and a wet coffee area with no obvious dry season. The annual mean rainfall, temperature, relative humidity, and solar radiation were 2,915 mm year^−1^, 22°C, 90.2%, and 15.9 MJ m^−2^ year^−1^, respectively.

There were 20 agroforestry systems with various shade types and management practices included in the partially randomized block design, where the shade trees played the major role, whereas subplots indicated the management practices (Supplementary Table S1). Three replicates were set up in the systems (Supplementary Figure S1). Shade type [originally 417 trees ha^−1^ (6 × 4 m^2^ interval)] was constituted by service tree and timber species that had different features (Supplementary Table S2).

Management practices included weed control, fertilization, and disease and pest control (Supplementary Table S3). *Coffea arabica* L. var. *Caturra* was planted at a density of 5,000 holes ha^−1^, and dead or dying plants were substituted annually. After planting (local practice), two plants within one hole were considered as one during the discussion. The inter-hole and inter-row distances were 1 and 2 m, respectively. The central part (225–300 m^2^) of the subplots (500–600 m^2^ in area) was researched (24 shade trees together with 100 coffee plants).

The coffee plants were manually pruned in 2004, with 1–4 resprouts left on every stump, based on the coffee resprout production potential during the subsequent harvest. Each coffee-planting hole constituted of 1–2 stumps and 1–4 resprouts per stump.

The coffee cherries from the different agroforestry systems were artificially harvested in the morning in both November 2013 and November 2014. All coffee cherries were then transported immediately from the field to the Industry Department of the Costa Rican Coffee Institute-ICAFE for wet processing and hot air drying. After that, all processed green coffee beans with vacuum packaging were shipped to the University of Nottingham, United Kingdom, and then stored at −20°C for analysis.

### 2.2. Physical and chemical analyses methodologies adopted for roasted and green coffee beans

#### 2.2.1. Coffee roasting

The coffee roasting process has been described by Caporaso et al. (11). Briefly, a coffee roaster (Roastilino, Fracino) with a nominal volume of approximately 200 g was used to roast the green coffee beans, and 50 g of each sample was prepared and roasted. The roasting time and air outlet temperature were set at 1 min 50 s and 166°C, respectively. After the first cracking process, all the coffee beans were roasted to a medium degree. Following the roasting, a mini chopper (CH180 mini chopper, 300 W, Kenwood) was used to grind 30 g of coffee beans into powdered form, and then the coffee powder samples were obtained using a 710-μm sieve. The samples were later kept under −20°C.

#### 2.2.2. Roasting loss determination

Green and roasted coffee beans were weighed with a balance (0.01 g, PFB 1200–2, KERN Ltd.) before and after roasting, and their weights were recorded (W_1_ for green beans and W_2_ for roasted beans). Thus,


(1) $$\text{Roasting loss}\;\left(g\right)=W_{1}\left(g\right)-W_{2}\left(g\right)$$

#### 2.2.3. Measurement of moisture content

All the coffee samples were weighed without containers and the weight was recorded as W_3_ (g). All the coffee samples were weighed together with their containers using a balance (0.01 g, PFB 1200–2, KERN Ltd.), and their total weight was recorded as W_4_ (g). Next, they were loaded into a 105°C drying oven (GenLab Ltd.). After 24 h, the coffee samples were weighed together with their containers again and their weight was recorded as W_5_ (g) (12). Thus:


(2) $$\text{Moisture content }\left(g/kg\right)=\frac{W_{4}\;\left(g\right)-W_{5}\left(g\right)\;}{W_{3}\left(g\right)}\times 1,000$$

#### 2.2.4. Measurement of green coffee bean density

The assumption was that all green coffee beans were considered to be half a triaxial ellipsoid (13). The length (major), width (minor), and thickness of each green coffee bean were measured using a Vernier-caliper (Accuracy: ± 0.02 mm, Carbon Fibre Composites Digital Caliper, Fisher Scientific) and recorded as a, b, and c, respectively. Thus, the volume of each green coffee bean could be calculated using the ellipsoid volume formula as follows:


(3) $$\text{The volume of individual bean }\left(mm^{3}\right)=\frac{1}{2}\times \frac{4}{3}\pi \frac{a}{2}\frac{b}{2}c=\frac{\pi abc}{6}\;$$

Ten green coffee beans (three replicates) were randomly selected from each plot and their length, width, and thickness were measured (14). After that, the volumes of each green coffee bean were calculated, and then, the average volume [V_a_ (mm^3^)] of the 10 green coffee beans was calculated. The total weight [W_6_ (g)] of the 10 green coffee beans was measured using a balance (0.01 g, PFB 1200–2, KERN Ltd.). Thus, the average density of an individual bean could be calculated as follows:


(4) $$\text{The average density of individual bean }\left(kg/m^{3}\right)=\frac{W_{6}\;\left(kg\right)}{V_{a}\;\left(m^{3}\right)}$$

#### 2.2.5. Measurement of bulk density

Approximately 20 g of the green coffee beans (three replicates) were weighed using a balance (0.01 g, PFB 1200–2, KERN Ltd., Fareham, United Kingdom). Next, the green coffee beans were put into a volumetric flask for volume measurement and recording (14). Thus, the average bulk density of the green coffee beans could be calculated.

#### 2.2.6. Measurement of mineral content

This study used acid digestion to prepare the sample and inductively-coupled plasma mass spectrometry (ICP-MS) to measure the mineral content of the green coffee beans (15).

For acid digestion, 0.2 g of the coffee powder was placed into plastic tubes provided by Microwave Digestion System (Microwave Digestion System: Multiwave Go, AutonPaar). Six milliliters of 70% nitric acid (Trace Analysis Grade, Fisher, UK) was put into the plastic tubes, and then placed in the microwave digestion system for 40 min with a heating program: the system temperature was increased from 0°C to 160°C over 15 min and then kept for 15 min under 160°C before a 10-min cooling until it reached ambient temperature. After incubation, the samples were transferred to glass bottles. Next, 4, 5, and 5 mL of distilled water were used sequentially to clean the plastic tubes; the used water was then transferred to glass bottles and combined. Finally, 2 mL of the samples were transferred to inductively-coupled plasma (ICP) tubes, followed by the addition of distilled water (8 mL).

ICP-MS (Thermo-Fisher iCAP-Q model, Thermo-Fisher Scientific, Bremen, Germany) was used to analyze multiple elements in dilutions. Three operational models were adopted for instrument operation, (i) one collision-cell (Q-cell) that used He showed kinetic energy differentiation (He-cell) for eliminating polyatomic interferences, (ii) a standard mode (STD) for the evaluation of collision cell, and (iii) a hydrogen mode (H_2_-cell) with H_2_ gas being the cell gas. The samples were introduced via the autosampler (Cetac ASX-520), integrating the PEEK nebulizer (Burgener Mira Mist) for the ASXpress fast absorption module. By using the ASXpress unit, the internal standards were introduced into the sample stream onto an isolated line, with Ir (5 μg L^−1^), Rh (10 μg L^−1^), and Ge (10 μg L^−1^) being incorporated into the 2% HNO_3_ (trace analysis grade; Fisher Scientific, United Kingdom). Approximately 0, 20, 40, and 100 μg L^−1^ of mineral solutions (including Ag, Al, As, Ba, Be, Cd, Ca, Co, Cr, Cs, Cu, Fe, K, Li, Mg, Mn, Mo, Na, Ni, P, Pb, Rb, S, Se, Sr., Tl, U, V, and Zn) (Claritas-PPT grade CLMS-2; SPEX Certiprep Inc., Metuchen, NJ, United States) were used as the external multifactor calibration criteria, respectively. Ca, Mg, Na, and K criteria within the scope 0–30 mg L^−1^ were created with the bespoke external multi-element calibration solution (PlasmaCAL, SCP Science, France). The calibration of P, B, and S adopted the in-house standards (H_3_BO_3_, KH_2_PO_4_, and K_2_SO_4_). The present study adopted in-sample switching for measuring P and B under the STD model and Se under the H_2_-cell model, whereas the remaining factors were under the He-cell mode. By adopting the Qtegra™ software (Thermo-Fisher Scientific), the obtained samples were processed using external cross-calibration between an analog detector and the pulse-counting models.

#### 2.2.7. Measurement of total lipid and fatty acid contents

The Bligh and Dyer (16) method was used to extract lipids from coffee. Coffee powders (0.1 g, wet basis) were soaked in chloroform:methanol (2:1; 2 mL) in a 15-mL centrifuge tube for 1 min with vortexing. Next, the samples were centrifuged at 1,600 × g and 4°C for 10 min. After centrifugation, three phases were presented in the samples; the bottom phase was carefully removed and placed in a clean glass vial. These steps were repeated thrice to fully extract the lipids. The bottom phase was then exposed to N_2_ to avoid lipid oxidation, and then stored. Finally, the lyophilized lipid samples were stored at −80°C with N_2_ added into the storage bottles to protect the lipid.

Approximately 0.1 g of coffee powder (wet foundation) was prepared, and the weight was recorded as W_7_ (g) using an analytical balance (0.1 mg, Sartorius 1712, Sartorius Ltd., Epsom, United Kingdom). An empty glass vial was weighed, and the weight was recorded as W_8_ (*g*). For each sample, the bottom phases of the three extractions were combined into the glass vial and dried using N_2_ gas. The weight of the glass vial containing the dried lipid extract was recorded as W_9_ (*g*) (17). The total lipid content by dry mass was calculated using the following formula:


(5) $$\text{The total lipid content }\left(dry\text{ mass}\right)\left(\frac{g}{kg}\right)=\frac{W_{9}\left(g\right)-W_{8}\left(g\right)}{W_{7}\left(g\right)\times \left(1-MC\right)}\times 1000\;$$

Where MC was moisture content as a percentage.

Approximately 0.1 g of methyl pentadecanoate was weighed, and dissolved in 10 mL chloroform. After being added to the dried lipid extract, chloroform (2 mL) was vortexed for 2 min. The mixture (1 mL) was filtered with the PTFE syringe filter (0.45 μm, Whatman, Buckinghamshire, United Kingdom), and moved into the novel glass vial. Internal reference (100 μL) was added, and then 200 μL of trimethylsulfonium hydroxide solution was added for the change of fatty acids to fatty acid methyl esters (FAMEs). The mixture was incubated for 10 min at room temperature to guarantee a complete conversion. Finally, the mixture was placed into a gas chromatography (GC) vial for analysis (17).

A gas chromatograph (Trace GC Ultra, Thermo Scientific) containing an auto-injection system (AS3000) and connected with a quadruple mass spectrometer (DSQII Quadrupole GC–MS, 50–380 m/z, Thermo Scientific) was used. A vaporization injector in the split model (3.0 mm liner, split flow 50 mL min^−1^, 250°C) was adopted to inject the sample (1 μL) in the capillary column (Phenomenex Zebron ZB-FFAP, film thickness 0.25 μm, 30 m × 0.25 mm I.D). For the oven, it was first kept under 120°C for 1 min before being heated at 5°C min^−1^ to 250°C for 2 min. FAME standards (Supelco37 Component FAME Mix) were adopted to compare the retention time of each peak. The Thermo Scientific Xcalibur software program was adopted to compare the mass spectrum with a standard library for identification verification. The Thermo Scientific Xcalibur software program was adopted to calculate the peak area of each FA. Moreover, the FA content was calculated using the formula below (17):


(6) $$\begin{array}{l} \text{Fatty acid concentration }\left(dry\text{ mass}\right)\;\;(mg/kg)\\ =\frac{\text{Peak area of fatty acid}\times \text{Amount of internal standard }\left(mg\right)}{\text{Peak area of internal standard}\times \text{Dry weight of coffee powder }\left(kg\right)}\; \end{array}$$

*(Dry weight of coffee powder = wet weight of coffee powder × (1-moisture content))*

#### 2.2.8. Measurement of caffeine content

The criteria for the preparation of caffeine was 10/20/40/50 mg L^−1^. After being put into a 50-mL centrifuge tube containing boiling water (20 mL), 0.1 g of coffee powder was vortexed for 5 min. The samples were centrifuged for 10 min at 1600 × g and at room temperature. After the centrifugation, the liquid stage was transferred to the novel glass vial. Caffeine was extracted completely by repeating the above processes three times. The mixed sample was then left for cooling under ambient temperature, followed by filtration using a 0.45-μm syringe filter (hydrophilic nylon syringe filter, Millipore Corporation). Before liquid chromatography-mass spectrometry, the eventual water extract dilution (1:1) was performed (LC/MS) (18).

The LC device (1,100 Series, Agilent) containing one degasser (G1322A, Agilent), an auto-sampler (G1313A, Agilent), and a pump (G1312A, Agilent) was connected to the Quattro Ultima mass spectrometer (Micromass, UK Ltd.) under the electrospray ion source.

Caffeine was separated with a 5-μm Kromasil 5 ODS (C18) column (250 × 3.20 mm, Phenomenex), and the column was stored at room temperature. The mobile stage contained 0.3% aqueous formic acid (eluent A) and methanol (eluent B) at the 0.4 mL min^−1^ flow rate. Next, 25% of B was applied first as a solvent, while 60% of B was modified immediately after this operation at 8 min. During the injection process, the column was re-equilibrated with 25% of B for 4 min.

Caffeine (M + H)^+^ ions were generated by operating the electrospray ionization source in the positive model (3.5KV) from 4.0–8.0 min with the 400°C desolvation temperature and 500 L h^−1^ desolvation gas (N_2_) flow rate. The caffeine measurement was then completed by operating a mass spectrometer under a selected ion recording model. The criteria for the molecular weight and retention time were compared to identify the compounds. Masslynx software (version 4.0, Waters) was adopted to compare the peak regions of the samples and the norms for quantification (18).

#### 2.2.9. Measurement of carbohydrate content

The carbohydrate standard mixture, comprising arabinose, galactose, glucose, and mannose was prepared. The concentration of arabinose and glucose were 2.5, 5, 10, and 20 mg L^−1^, while the concentration of galactose was 5, 10, 40, and 80 mg L^−1^, and the concentration of mannose was 5, 10, 20, and 40 mg L^−1^.

Saeman’s hydrolysis was used to extract carbohydrates from green coffee beans (19). Approximately 30 mg of green coffee powder (wet basis) was put in a round bottom Pyrex tube, and 1 mL of 12 M H_2_SO_4_ was added. The tube was sealed and put into a 37°C water bath for 60 min. The samples were then eliminated, and 11 mL of demineralized water was added. The samples were then put into a 100°C water bath for 120 min. After that, the samples were removed and placed in a cold-water bath until they were cool enough to handle. Next, 1 mL of the sample was then placed in a 15-mL Falcon tube, followed by the addition of 10 mM NaOH (9 mL) to make a 1–10 dilution.

Carbohydrates were analyzed using the Dionex ICS-3000 Ion Chromatography System (Thermo Scientific). The reagent-free ion chromatography was configured with an auto-injection system (AS autosampler) and coupled to the Dionex ICS-3000 system, with a computer controller, and electrochemical detection (ED 40). The mobile stage (10 mM NaOH) was separated using the CarboPac PA 20 column (3 × 150 mm) at the column temperature, injection volume, and flow rate of 30°C, 10 μL, and 0.5 mL min^−1^, respectively. Their retention time with standard solutions was compared to identify the carbohydrates. The peak areas were calculated using Thermo Scientific Chromeleon software. The concentration of the carbohydrates was determined using a standard curve.

### 2.3. Statistical analysis

In this experiment, shade types were used as the major plot, whereas the subplots indicated management practices and the imbalanced framework was observed because not every management practice was indicated for every shade type (Supplementary Table S4). Hence, diversities between various management practices and shade types were compared with the given pre-planned contrast models (5, 20) (Supplementary Table S4).

Adopting SPSS statistic software (Version 22.0, IBM, United States), the one-way analysis of variance contrast model was used to analyze the data, with treatments assigned as the contrast element for exploring eight contrast groups (Supplementary Table S4). The significant differences between the contrast groups are presented as *p* < 0.05. Moreover, the mean for each management practice together with the shade type in every contrast model was calculated.

## 3. Results and discussion

### 3.1. Influences of fertilizer degrees and shade types on the physical attributes of green coffee bean

According to the contrast model, contrast results for density, bulk density, moisture content, and roasting loss were determined (Table 1). In terms of density, bulk density, moisture content, and roasting loss, there were no significant differences (*p* > 0.05) between the management practices.

**Table 1:** Contrast results for density (Kg/m^3^), bulk density (Kg/m^3^), moisture content (g/Kg) and roasting loss (g/Kg).

Contrast	Density (kg/m^3^)			Bulk Density (kg/m^3^)			Moisture content (g/Kg)			Roasting loss (g/kg)		
Managements	Mean	Std.	*p*-value	Mean	Std.	*p*-value	Mean	Std.	*p*-value	Mean	Std.	*p*-value
IC vs MC	1372	78	0.184	671	19	0.531	110	9	0.691	130	9	0.874
	1399			668			111			130		
MC vs IO	1363	95	0.399	669	24	0.362	112	11	0.281	131	11	0.207
	1376			673			110			128		
IO vs LO	1399	55	0.312	670	14	0.891	109	7	0.462	131	6	0.236
	1371			671			112			127		
IC vs IO	1372	67	0.587	670	17	0.941	111	8	0.337	132	8	0.057
	1385			670			108			127		
Shade types												
FS vs S	1462	35	**0.018**	672	34	0.495	108	5	**0.033**	125	6	**0.017**
	1372			668			111			132		
E vs FS	1363	25	**0.024**	667	14	0.503	114	2	**0.002**	135	3	**0.003**
	1462			672			108			125		
Ser. vs Tim.	1381	35	0.335	665	34	0.159	110	5	**0.028**	127	6	**0.002**
	1359			674			113			135		
LT vs NLT	1371	25	0.806	681	14	0.054	111	2	0.276	126	3	0.254
	1364			668			107			122		

Values were shown as mean, standard deviation (Std.) as well as diversity significance *p* < 0.05 were displayed in bold. IC, Intensive Conventional; MC, Moderate Conventional; IO, Intensive Organic; LO, Low Organic; FS, Full Sun; S, Shaded; E, *Erythrina poepiggiana*; Ser., Service; Tim, Timber; LT, Legume Timber; NLT, Non-Legume Timber.

The density of green coffee beans was approximately 1,300–1,400 kg m^−3^, which was higher than the water density (1,000 kg m^−3^) (13). Consequently, the green coffee bean density increased with decreasing moisture content. Therefore, under the full sun system, the green coffee bean density significantly increased (*p* < 0.05) compared with the shaded and *Erythrina* systems. However, for the service and timber groups, no significant difference (*p* > 0.05) was observed in green coffee bean density, although the green coffee bean moisture content significantly decreased (*p* < 0.05) under the service system compared with the timber system. This might be reasonable as shading also prolongs the ripening of coffee beans to help the coffee plant absorb more nutrients. Under a similar shaded system, on the one hand, shading increases the moisture content of green coffee beans, which leads to a decrease in coffee bean density; on the other hand, shading improves the absorbance of nutrients, which may increase coffee bean density (21). Under a similar shaded system, these two factors compete with each other during the growth of the coffee plant, resulting in the density of green coffee beans showing no significant difference (*p* > 0.05) between the service and timber systems.

With regards to the moisture content, the green coffee beans under the full sun system significantly decreased (*p* < 0.05) compared with the *Erythrina* and shaded systems. Moreover, under the service system, green coffee beans had significantly decreased (*p* < 0.05) moisture content compared with that under the timber system. Compared with the full sun system, the major impact of the shaded system on coffee physiology is related to the reduced wind speeds and temperature fluctuation, which led to the increase in air relative humidity and variations in the aerodynamic roughness of the coffee plants. Taken together, these changes decrease leaf-to-air vapor pressure deficit and restrain the stomatal opening, which results in a decline in transpiration rates. Therefore, water loss caused by exceeding coffee tree evapotranspiration will decrease (1). In addition, the shaded agroforestry system improves the water status of the coffee tree, which is known to affect bean size and density (1). The size of ripe cherries and coffee beans increases in the shaded system because the shading decreases air and plant surface temperature, and thus prolongs bean ripening to generate larger fruits.

During the coffee bean roasting process, even though dry matter loss was approximately 5–8%, the total roasting loss was inconceivably approximately 15–18% (22). Furthermore, the water content decreased from approximately 10–12% to 2–3%, when bean temperatures were below 160°C (23). These indicated that the water loss contributed to the main mass loss during coffee bean roasting. Consequently, the roasting loss of green coffee beans in the full sun system was reduced significantly (*p* < 0.05) compared with the *Erythrina* and shaded systems because they presented lower moisture content in the full sun system. Similarly, it can be observed that the roasting loss of green coffee beans in the service system was significantly downregulated (*p* < 0.05) relative to that in the timber system.

### 3.2. Roles of fertilizer degrees and shade types in green coffee bean mineral contents

Twenty-eight minerals, including macroelements, such as K, Mg, P, S, and Ca, and microelements, such as Mn, Fe, Cu, Zn, Na, Mo, Co, Ba, Sr., and Rb, were found in the green coffee beans, while only some main minerals (K, Mg, P, S, Ca, Mn, Fe, Cu, Zn, Na, and Mo) were analyzed and are presented in Table 2. No significant differences (*p* > 0.05) were detected in Mg and Fe contents under the various management practices and shade types.

**Table 2:** Contrast results for major mineral contents (mg/g) by dry weight of green coffee beans.

Contrast	K (g/Kg)			Mg (g/Kg)			P (g/Kg)			S (g/Kg)			Ca (g/Kg)		
Managements	Mean	Std.	*p*-value	Mean	Std.	*p*-value	Mean	Std.	*p*-value	Mean	Std.	*p*-value	Mean	Std.	*p*-value
IC vs MC	20	1.4	0.292	2.0	0.2	0.651	1.8	0.09	0.251	1.9	0.13	0.088	1.4	0.10	0.059
	20			2.0			1.8			1.8			1.4		
MC vs IO	19	0.7	**<0.001**	2.1	0.2	0.813	1.8	0.08	**<0.001**	1.8	0.16	0.578	1.4	0.13	**<0.001**
	20			2.1			1.9			1.8			1.6		
IO vs LO	20	1.0	0.676	2.1	0.1	0.699	1.9	0.06	0.98	1.8	0.09	0.26	1.6	0.07	**0.003**
	20			2.1			1.9			1.8			1.5		
IC vs IO	20	1.2	0.579	2.0	0.1	0.145	1.8	0.08	**<0.001**	1.8	0.11	0.151	1.4	0.09	**<0.001**
	20			2.1			1.9			1.8			1.6		
Shade types															
FS vs S	20	2.5	0.323	2.1	0.3	0.205	1.8	0.15	0.654	1.9	0.23	0.283	1.5	0.08	**0.002**
	20			2.0			1.8			1.8			1.4		
E vs FS	19	1.0	0.086	2.0	0.1	0.266	1.7	0.06	0.758	1.7	0.09	**0.003**	1.3	0.07	**<0.001**
	20			2.1			1.8			1.9			1.5		
Ser. vs Tim.	19	2.5	0.476	2.1	0.3	0.844	1.8	0.15	0.692	1.7	0.23	0.08	1.5	0.08	0.768
	20			2.1			1.8			1.8			1.5		
LT vs NLT	20	1.0	0.658	2.1	0.1	0.551	1.8	0.06	0.247	1.8	0.09	0.054	1.4	0.07	0.052
	20			2.0			1.8			1.7			1.5		

Contrast	Mn (mg/Kg)			Fe (mg/Kg)			Cu (mg/Kg)			Zn (mg/Kg)			Na (mg/Kg)			Mo (μg/kg)		
Managements	Mean	Std.	*p*-value	Mean	Std.	*p*-value	Mean	Std.	*p*-value	Mean	Std.	*p*-value	Mean	Std.	*p*-value	Mean	Std.	*p*-value
IC vs MC	70	3.7	**<0.001**	33	5.2	0.957	17	2.3	0.052	7	0.7	**<0.001**	5.1	0.7	0.711	55	18	0.578
	47			32			15			6			5.2			57		
MC vs IO	47	4.5	**<0.001**	33	6.3	0.749	16	2.8	0.599	6	1.4	0.082	5.4	0.8	**<0.001**	54	22	**<0.001**
	22			34			16			6			4.9			220		
IO vs LO	22	2.6	0.134	30	3.7	0.231	15	1.6	0.342	6	0.8	0.671	4.8	0.5	**0.024**	200	13	**<0.001**
	24			32			16			6			5.4			142		
IC vs IO	62	3.2	**<0.001**	32	4.5	0.197	17	1.0	**0.008**	7	0.8	**<0.001**	4.9	0.6	0.874	54	16	**<0.001**
	22			30			15			6			4.9			215		
Shade types																		
FS vs S	69	6.3	**<0.001**	33	9.0	0.587	15	0.9	**0.006**	7	1.9	0.549	4.5	1.2	0.397	58	31	0.651
	55			32			16			7			4.4			55		
E vs FS	50	2.6	**<0.001**	31	3.7	0.299	18	1.6	**<0.001**	8	0.8	**0.023**	5.2	0.5	**0.002**	53	13	0.418
	69			33			15			7			4.5			58		
Ser. vs Tim.	35	6.3	0.406	32	9.0	0.381	17	0.9	0.205	6	1.9	0.162	4.8	1.2	0.096	142	31	0.07
	34			34			16			6			5.2			147		
LT vs NLT	34	2.6	0.283	35	3.7	0.223	15	1.6	0.772	6	0.8	0.951	5.0	0.5	0.728	160	13	0.105
	35			32			15			6			5.1			155		

Values were shown as mean, standard deviation (Std.) as well as diversity significance *p* < 0.05 were displayed in bold. IC, Intensive Conventional; MC, Moderate Conventional; IO, Intensive Organic; LO, Low Organic; FS, Full Sun; S, Shaded; E, *Erythrina poepiggiana*; Ser., Service; Tim, Timber; LT, Legume Timber; NLT, Non-Legume Timbe.

The ability of a plant to absorb minerals and other nutrients from the soil is extremely complicated. It is determined by the gene as well as the growing environment of the plant, such as temperature, light intensity, fertilizer level, moisture content, and salinity of the soil (24).

In this experiment, the greater quantities of K and P in green coffee beans under the intensive organic (IO) management practice compared with the intensive conventional (IC) and moderate conventional (MC) management practices were related to the higher input of these elements in the IO management practice. However, in terms of IO and LO management practices, although the application amounts of K and P in the IO (326 and 205 kg ha^−1^ year^−1^) were much higher than those in the LO management practice, which were 44 kg ha^−1^ year^−1^ and 2 kg ha^−1^ year^−1^ for K and P, respectively, no significant differences (*p* > 0.05) in K and P contents of green coffee beans were detected between these two management practices. Haggar et al. (5) reported that the organic management practice improved the organic matter content and cation exchange capacity of the soil in the Costa Rica site. Therefore, although the application amounts of K and P in the LO management practice were much lower, the LO management practice was able to make up for the low input amounts with more organic matter and a stronger cation exchange capacity.

The higher Ca content of green coffee beans in the IO management practice was related to the high content of Ca in chicken manure (25). In this experiment, chicken manure was used as the fertilizer in the IO management practice. Additionally, the Mn, Cu, and Zn contents of green coffee beans decreased significantly (*p* < 0.05) in the IO, while the Mo quantity was significantly higher (*p* < 0.05) in the enhanced IO management practice, as organic management practice decreased the soil acidity or increased soil pH (5), and thus, restricted the absorptivity of Mn, Cu, and Zn, and improved the absorbance of Mo (26). For Na, its content was significantly lower (*p* < 0.05) under the IO system compared with the MC and LO systems, as the higher absorbance of Ca in the IO system restrained Na uptake (6).

The moisture content of the soil, light intensity, and temperature are the major external elements that influence the absorption and transport of minerals in the plant (27). The Costa Rica site is regarded as a wet coffee zone with an average annual rainfall of 2,915 mm year^−1^. Thus, all coffee plants and corresponding soils in the different shade types have no shortage of water and the moisture content cannot be considered a factor to affect the mineral absorption of coffee plants from the soil. The shade system reduces the temperature and light intensity of coffee plants as well as the soil (1). Nonetheless, the ability of plants to absorb minerals increases with increasing temperature when the temperature is below 40°C (28). Moreover, the higher light intensity improves the absorptivity of the minerals in plants (27). Therefore, the S, Ca, and Mn contents were significantly upregulated (*p* < 0.05) under the full sun system compared with those under the *Erythrina* and shaded systems because of the elevated temperature as well as enhanced light intensity. However, the quantities of Cu, Zn, and Na significantly decreased (*p* < 0.05) in the full sun system compared with those of the *Erythrina* and shaded systems because of the antagonism of Ca, Cu, Zn, and Na (6). Compared with Cu, Zn, and Na uptakes, the coffee plant absorbed much more Ca, therefore, the excess Ca uptake restricted the coffee plant from uptaking more Cu, Zn, and Na.

### 3.3. Roles of fertilizer degrees together with shade types in the total lipid and fatty acid quantities of green coffee beans

Total lipid and FA levels were measured, and 10 fatty acids, including palmitic acid (C16:0), linoleic acid (C18:2), stearic acid (C18:0), arachidic acid (C20:0), behenic acid (C22:0), oleic acid (C18:1), lignoceric acid (C24:0), linolenic acid (C18:3), cosenoic acid (20:1), and myristic acid (C14:0) were found in the green coffee beans (Table 3).

**Table 3:** Contrast outcomes for total lipid and fatty acids contents (g/Kg and mg/Kg) based on green coffee bean dry weight.

Contrast	Total lipid (g/Kg)			Palmitic acid (g/Kg)			Linoleic acid (g/Kg)			Stearic acid (g/Kg)			Arachidic acid (g/Kg)		
Managements	Mean	Std.	*p*-value	Mean	Std.	*p*-value	Mean	Std.	*p*-value	Mean	Std.	*p*-value	Mean	Std.	*p*-value
IC vs MC	163	8	**0.021**	37	0.5	0.368	14	0.2	0.635	10.3	2.7	0.592	8.9	1.8	0.729
	151			36			14			9.9			8.8		
MC vs IO	149	6	**0.003**	35	0.6	**0.033**	13	0.9	0.134	8.9	3.3	0.153	8.4	2.2	0.522
	162			37			14			9.7			8.6		
IO vs LO	147	9	0.428	36	0.3	0.229	14	1.5	0.516	9.8	1.9	0.717	9.1	1.3	0.771
	142			38			14			10.1			8.9		
IC vs IO	152	12	0.3	36	0.4	0.35	14	1.8	0.318	10.2	2.4	0.231	9.0	1.6	0.512
	146			35			13			9.3			8.7		
Shade types															
FS vs S	151	18	0.195	35	0.8	0.114	13	0.4	0.1	9.4	1.7	0.262	8.3	1.2	0.146
	159			37			14			10.3			9.1		
E vs FS	173	14	**<0.001**	41	1.2	**<0.001**	16	0.2	**0.001**	13.0	1.9	**<0.001**	11.0	1.3	**<0.001**
	151			35			13			9.4			8.3		
Ser. vs Tim.	167	18	**0.001**	39	0.8	**0.002**	15	0.4	**0.001**	11.9	1.7	**<0.001**	10.4	1.2	**<0.001**
	146			35			13			8.8			8.2		
LT vs NLT	155	14	0.59	37	1.2	0.267	14	0.2	0.39	9.0	1.9	0.789	8.4	1.3	0.869
	151			35			13			9.3			8.3		

Contrast	Behenic acid (g/Kg)			Oleic acid (g/Kg)			Lignoceric acid (g/Kg)			Linolenic acid (mg/Kg)			Cosenoic acid (mg/Kg)			Myristic acid (mg/kg)		
Managements	Mean	Std.	*p*-value	Mean	Std.	*p*-value	Mean	Std.	*p*-value	Mean	Std.	*p*-value	Mean	Std.	*p*-value	Mean	Std.	*p*-value
IC vs MC	1.9	0.5	0.849	0.9	0.2	0.449	0.71	0.2	0.714	138	33	0.308	43	9	0.247	28	10	0.507
	1.9			0.9			0.70			130			40			28		
MC vs IO	1.8	0.6	0.734	0.8	0.3	**<0.001**	0.67	0.2	0.797	133	40	0.868	38	11	**0.003**	28	12	0.836
	1.8			1.0			0.68			134			44			28		
IO vs LO	2.0	0.3	0.245	0.9	0.2	0.475	0.77	0.1	0.3	140	23	0.689	41	7	0.152	30	7	0.261
	1.8			0.9			0.71			144			45			26		
IC vs IO	1.9	0.4	0.603	0.9	0.2	0.07	0.71	0.1	0.812	132	28	0.496	40	8	0.521	30	9	0.531
	1.8			0.8			0.70			138			39			28		
Shade types																		
FS vs S	1.8	0.8	0.265	0.9	0.1	0.229	0.66	0.2	0.2	124	27	0.165	39	6	0.195	24	11	0.114
	1.9			0.9			0.72			137			42			28		
E vs FS	2.4	0.3	**0.001**	1.2	0.2	**<0.001**	0.90	0.1	**<0.001**	169	13	**<0.001**	51	7	**<0.001**	34	5	**0.006**
	1.8			0.9			0.66			124			39			24		
Ser. vs Tim.	2.3	0.8	**<0.001**	1.1	0.1	**0.002**	0.90	0.2	**<0.001**	162	27	**<0.001**	47	6	**0.024**	30	11	0.597
	1.7			0.9			0.64			128			41			28		
LT vs NLT	1.8	0.3	0.614	0.9	0.2	0.567	0.67	0.1	0.64	134	13	0.817	42	7	0.276	33	5	0.057
	1.7			0.9			0.64			137			39			26		

Values were shown as mean, standard deviation (Std.) as well as diversity significance *p* < 0.05 were displayed in bold. IC, Intensive Conventional; MC, Moderate Conventional; IO, Intensive Organic; LO, Low Organic; FS, Full Sun; S, Shaded; E, *Erythrina poepiggiana*; Ser., Service; Tim, Timber; LT, Legume Timber; NLT, Non-Legume Timbe.

The formation of lipids in coffee plants is a complicated process, which includes many complex biochemical reactions, especially enzymatic reactions. Furthermore, energy and some chemical elements, such as C, H, O, N, and P play an essential role in lipid biosynthesis in the coffee plant (29). Ohlrogge and Browse (29) stated that the acetyl-coenzyme A (CoA) in the plastid contributed to all the carbon atoms present in FA, and they also explained all possible lipid biosynthesis pathways in the plant. In addition, they considered that the synthesis rate of FA in leaves was six times higher in the light than in the dark. As a result, in this experiment, various fertilizer degrees and shade types (light intensity) might affect the total lipid and FAs quantities in green coffee beans.

Regarding the various fertilizer degrees, the overall lipid content was significantly reduced (*p* < 0.05) in the MC compared with the IC management practice due to the lower input amount of fertilizer in the MC management practice. With the slower release of plant-available nutrients and lower availability of organic fertilizers in the IO management practice and even a higher fertilizer input level (30), the overall lipid and all FA quantities under the IO were similar to those under the IC management practice and even significantly higher (*p* < 0.05) for the overall lipid and a few FAs, including oleic acid, palmitic acid, and cosenoic acid than under the MC management practice. Therefore, for total lipid and FA contents in the green coffee beans, the performance of the IO management practice with a higher fertilizer input amount kept up with its counterpart IC, and to some extent, even exceeded the MC management practice, as the IO management practice improved the soil environment, and increased cation exchange capacity and organic matter in the soil (5). Furthermore, for the IO and LO, although the input amount of fertilizer in the LO was much lower, no significant difference (*p* > 0.05) was detected in the total lipid and FAs contents between these two organic management practices. In our contrast model, while comparing with the IO, the LO management practice was integrated with legume species *Chloroleucon* and *Erythrina*. Typically, legume species supplement N_2_ shortage because of the low input degree by N_2_ fixation (31). In contrast, the lower external inputs may be compensated by the high pruning intensity such as in *Erythrina*, due to the abandonment of *Erythrina* shade tree-pruned branches and leaves (5).

With regards to the role of shade types in the overall lipid and FA contents, there were no significant differences (*p* > 0.05) between the full sun and shaded systems, and between non-legume and legume timber systems, proving that neither N_2_ fixation nor light intensity was a major element affecting lipid levels in this experiment. Nonetheless, the overall lipid and FA quantities were significantly upregulated (*p* < 0.05) in the *Erythrina* system compared with the full sun and timber systems (the *Erythrina* was the only service shade tree in the current experiment, thus, in the contrast group service vs. timber, the service tree could be replaced directly with *Erythrina*). It could be explained by the intensive pruning of *Erythrina* (7). The intensive pruning of *Erythrina* supplied available nutrients directly to the soil as well as to the coffee plant, which could promote the biosynthesis of lipids in the coffee plant.

### 3.4. Influence of fertilizer degrees and shade types on the carbohydrates and caffeine contents of green coffee bean

The polysaccharides content was decided by measuring the acidolysis solution of coffee powder using high-performance liquid chromatography (HPLC). Table 4 shows the result of caffeine and polysaccharides content.

**Table 4:** Contrast outcomes for caffeine (g/Kg) and carbohydrates contents (g/Kg) based on green coffee bean dry weight.

Contrast	Caffeine (g/Kg)			Galactose (g/Kg)			Mannose (g/Kg)			Glucose (g/Kg)			Arabinose (g/Kg)		
Managements	Mean	Std.	*p*-value	Mean	Std.	*p*-value	Mean	Std.	*p*-value	Mean	Std.	*p*-value	Mean	Std.	*p*-value
IC vs MC	12.2	1.1	**0.003**	219	37	0.844	109	17	0.892	69	9	0.828	33.9	4	0.945
	11.3			217			108			69			33.8		
MC vs IO	11.8	1.4	**0.01**	215	45	0.779	107	21	0.41	67	12	0.525	33.7	5	0.947
	12.4			217			111			69			33.8		
IO vs LO	12.3	0.8	0.681	212	26	0.622	103	12	0.47	67	7	0.899	33.9	3	0.867
	12.1			218			108			66			34.1		
IC vs IO	12.4	1.0	0.802	223	32	0.769	111	15	0.389	70	8	0.689	34.4	3	0.983
	12.3			220			107			69			34.3		
Shade types															
FS vs S	12.0	2.0	**0.014**	181	64	0.287	101	29	0.037	55	16	0.08	32.7	7	0.173
	11.1			191			111			65			34.2		
E vs FS	11.7	0.8	0.539	222	26	**0.002**	125	12	**<0.001**	70	7	**<0.001**	37.1	3	**0.002**
	12.0			181			101			55			32.7		
Ser. vs Tim.	11.7	2.0	0.405	231	64	0.222	114	29	0.332	74	16	0.053	35.5	7	0.132
	12.0			218			109			69			33.8		
LT vs NLT	12.2	0.8	0.134	217	26	0.507	109	12	0.599	68	7	0.357	33.9	3	0.701
	11.5			226			113			71			34.4		

Values were shown as mean, standard deviation (Std.) as well as diversity significance *p* < 0.05 were displayed in bold. IC, Intensive Conventional; MC, Moderate Conventional; IO, Intensive Organic; LO, Low Organic; FS, Full Sun; S, Shaded; E, *Erythrina poepiggiana*; Ser., Service; Tim, Timber; LT, Legume Timber; NLT, Non-Legume Timber.

In plants, the conversion of inorganic C into organic C is accomplished mainly by photosynthesis. Otherwise, plants can take the fungi-fixed and organic-derived C (32). Moreover, light intensity, water availability, CO_2_ concentration, and temperature affect photosynthesis (33). For every contrast group under the various management practices, shade types were kept constant. Therefore, temperature and light intensity might remain unchanged when various management practices were compared for every contrast group. The various management practices did not affect CO_2_ content because the atmospheric condition was identical. In addition, the Costa Rica site represented a wet coffee area with abundant water resources. Hence, carbohydrate contents were not significant (*p* > 0.05) among the various management practices.

No significant differences (*p* > 0.05) were observed with regard to carbohydrate content in the full sun compared with the shaded systems, or in the service compared with timber, as well as in non-legume compared with legume timber systems in this experiment, indicating that neither N_2_ fixation nor shaded cover or light intensity of the shade trees was a major element affecting carbohydrate content in the green coffee beans. However, the galactose, mannose, glucose, and arabinose contents were significantly higher (*p* < 0.05) under the *Erythrina* system when compared with the full sun system. This was related to the high organic matter, especially the organic C used directly by the coffee plant due to the extreme pruning of the *Erythrina* shade tree (the branches and leaves of *Erythrina* were maintained on the field after pruning) (34).

The caffeine quantity was significantly enhanced (*p* < 0.05) in the IC as well as IO management practices compared with the MC because of the increased fertilizer amounts, particularly nitrogen. Following the long-term management practice, the IO management practice showed a similar green coffee bean caffeine level compared with the IC management practice, which might greatly increase (*p* < 0.05) compared with the MC management practice. However, the caffeine level was not significantly different (*p* > 0.05) in the IO as well as the LO systems. While relative to the IO management practice, the LO management practice was integrated with *Chloroleucon* and *Erythrina*, two legume species, which supplement the N_2_ shortage because of the low input degree by N_2_ fixation (10). In contrast, the lower external inputs may be compensated by the high pruning intensity such as in *Erythrina*, due to the elimination of *Erythrina* shade tree-pruned branches and leaves (7, 34).

The caffeine quantity in the green coffee beans significantly increased (*p* < 0.05) in the full sun system relative to the shaded system, as the light improved the formation of the purine ring of caffeine (22). Nevertheless, the green coffee bean caffeine quantity did not show any significant differences (*p* > 0.05) between legume timber and non-legume timber systems, even though the legume trees could fix N_2_, which is the main component of caffeine. Furthermore, there were no significant differences (*p* > 0.05) in green coffee bean caffeine contents between the *Erythrina* and full sun systems, or between the timber and service systems, although shade cover affecting the light intensity between these shade systems was significantly different. As a result, the N_2_ fixation in the legume tree and the differences in shade cover between the *Erythrina* and full sun systems, or between the timber and service systems were not major elements to influence the caffeine quantity in the green coffee beans.

To improve the responses of agroforestry systems in relation to the physical and chemical characteristics of the coffee bean, the entry of adequate light must be controlled with combinations, densities, and adequate management practices of trees associated with coffee cultivation. The *Erythrina* service tree combined with timber or alone, can generate contributions from the annual pruning practice with the incorporation of biomass, in addition to N_2_ fixation. The inclusion of organic fertilizers in fertilization programs constitutes a strategy with potential.

## 4. Conclusion

In conclusion, compared with conventional management practices, the IO management practice improves the nutritional content of coffee beans, such as some minerals, lipids, and caffeine. Therefore, the IO management practice can be considered an excellent substitution for the MC management practice. Additionally, to some extent, the IO management practice may be a good option, when there is a cost limitation of the IC management practice. In terms of shade types, the *Erythrina* system promotes the performance of green coffee beans due to the replenishment of organic matter in the soil by extremely strong pruning. As a result, the *Erythrina* system can be used as the main shade tree.

## Data availability statement

The original contributions presented in the study are included in the article/Supplementary material, further inquiries can be directed to the corresponding author.

## Author contributions

SX: methodology, data curation, formal analysis, investigation, writing–original draft, and writing–review and editing. YL and GC: data curation and writing–original draft. ZS: formal analysis, and writing–review and editing. FM: writing–original draft, and writing–review and editing. NY: supervision, and writing–review and editing. EM: methodology, project administration, and resources. IF: methodology, conceptualization, project administration, and supervision. All authors contributed to the article and approved the submitted version.

## Funding

This work was supported by Guizhou Provincial Science and Technology Projects (QKHPTRC-CXTD [2022] 002, QKHZC [2022] ZD No. 015, and QKHJC-ZK [2021] YB 175); the BBSRC [BB/N021126/1]; Talent Introduction Program of Guiyang University (GYU-ZRD [2018]-022); the Discipline and Master’s Site Construction Project of Guiyang University by Guiyang City Financial Support Guiyang University [SY-2020]; and the Innovation and Entrepreneurship Project of Guizhou for College Students (S202110976077).

## Conflict of interest

The authors declare that the research was conducted in the absence of any commercial or financial relationships that could be construed as a potential conflict of interest.

## Publisher’s note

All claims expressed in this article are solely those of the authors and do not necessarily represent those of their affiliated organizations, or those of the publisher, the editors and the reviewers. Any product that may be evaluated in this article, or claim that may be made by its manufacturer, is not guaranteed or endorsed by the publisher.

## Figures and Tables

**Table:** 

ANOVA	analysis of variance
C	*Chloroleucon Eurycyclum*
CATIE	Centro Agronómico Tropical de Investigación y Enseñanza, Tropical Agricultural Research and Higher Education Center
CE	*Chloroleucon Eurycyclum* and *Erythrina Poepiggiana*
CoA	acetyl-coenzyme A
CT	*Chloroleucon Eurycyclum* and *Terminalia Amazonia*
E	*Erythrina Poepiggiana*
ED	electrochemical detection
ET	*Erythrina Poepiggiana* and *Terminalia Amazonia*
FAMEs	fatty acid methyl esters
FS	Ffull sun
GC–MS	gas chromatograph mass spectrometry
HPLC	high-performance liquid chromatograph;
IC	intensive conventional
ICP-MS	inductively coupled plasma mass spectrometry
IO	intensive organic
LC–MS	liquid chromatograph mass spectrometry
LO	low organic
MC	moderate conventional
Std.	standard deviation
S	shaded system
Ser.	service shade tree system
SIR	selected ion recording
STD	standard mode
T	*Terminalia Amazonia*
Tim	timber shade tree system
