# Supplementary material for: Effects of agro-forestry systems on the physical and chemical characteristics of green coffee beans
Source: Front Nutr. 2023 Jul 12;10:1198802. doi: 10.3389/fnut.2023.1198802 (PMC10369047; doi:10.3389/fnut.2023.1198802)
Supplement: Supplementary file 1 [file Data_Sheet_1.docx]

**Supporting Information**

Effects of agro-forestry systems on the physical and chemical characteristics of green coffee beans

**Su Xu^1,2^****^*^, Yuze Liu^1^, Zhenchun Sun^2^, Guangjing Chen^1^, Fengwei Ma^1^, Ni Yang^2^, Elias de Melo Virginio Filho^3^, Ian D. Fisk^2^**

^1^ Food and Pharmaceutical Engineering Institute, Guiyang University, Guiyang 550005, Guizhou, People’s Republic of China

^2^ Division of Food Sciences, University of Nottingham, Sutton Bonington Campus, Loughborough, Leicestershire, LE12 5RD, UK.

^3^ Centro Agronómico Tropical de Investigación y Enseñanza, CATIE, Turrialba 7170, Costa Rica

* Correspondence:

Su Xu

[xs8515@126.com](mailto:Ian.Fisk@nottingham.ac.uk)

Twenty agro-forestry systems consisting of seven combinations of shade types and four types of management practices were used to treat the coffee crops in the field (Table 1). In terms of shade types, three kinds of shade trees including *Erythrina poepiggiana, Chloroleucon eurycyclum,* and *Terminalia amazonia* were selected according to the local habits (Table 2). With regards to the management practices, there were divided into two types including the conventional fertilizer (chemical fertilizer) and the organic fertilizer (chicken manure and coffee pulp). In addition, for the conventional fertilizer, the intensive conventional (IC) fertilizer and the moderate conventional (MC) fertilizer were used, while for the organic fertilizer, the intensive organic (IO) fertilizer and the low organic (LO) fertilizer were used. Table 3 shows the different fertilizer levels, weed controls, and disease/pest controls for each management practice.

**Table S1** Agroforestry systems under major plot (Shade type) together with subplot (Management) treatments (5)

| Shade types* | E | T | C | C+T | E+T | C+E | Full Sun |
| --- | --- | --- | --- | --- | --- | --- | --- |
| Management** | IC | IC |  |  |  | IC | IC |
|  | MC | MC | MC | MC | MC | MC | MC |
|  | IO | IO | IO | IO | IO | IO |  |
|  | LO | LO |  |  |  | LO |  |

*E: *Erythrina poepiggiana*; T: *Terminalia amazonia*; C: *Chloroleucon eurycyclum*;

**IC: Intensive conventional, MC: Moderate conventional, IO: Intensive organic, LO: Low organic; (n=3)

**Table S2** Shade tree features (5, 20)

| Shade tree species | Phenology | Canopy | N-fixer | Application |
| --- | --- | --- | --- | --- |
| *Erythrina poepiggiana* (E) | Evergreen | Low compact | Yes | Service |
| *Chloroleucon eurycyclum* (C) | Deciduous * | High spreading | Yes | Timber |
| *Terminalia amazonia* (T) | Deciduous * | High compact | No | Timber |

* Deciduous for approximately 20-30 days/year

**Table S3** Average fertilizer input degrees (Kg ha^-1^year^-1^) and weed/disease prevention from 2006 (5, 20)

| Management | Fertilization  N:P:K ** | Weed control | Disease/Pest control |
| --- | --- | --- | --- |
| IC | 287:20:150 | 6*  Herbicides | 3-4*  Fungicides/  Insecticides |
| MC | 150:10:75 | 5  Herbicides  4  Manual | 1-4  Fungicides/  Insecticides  as required |
| IO | 248:205:326 | 4  Manual | Organic substances as required |
| LO | 66:2:44 | 4  Manual | No |

IC: Intensive conventional, MC: Moderate conventional, IO: Intensive organic, LO: Low organic;

* Treatment number adopted annually.

** Fertilization degrees (Kg ha^-1^year^-1^) indicate the average values within a 7-year period (2003-2009); LO systems received an identical fertilization to IO system during 2004-2006, because of site restrictions not permitting efficient setting up of organic coffee in the presence of decrease inputs. IO fertilization: chicken manure 10 t ha^-1^year^-1^and K-Mag (fertilizer)100kg ha^-1^year^-1^; LO fertilization: coffee pulp 5 t ha^-1^year^-1^

**Table S4** Main contrasts adopted for analyzing Shade type and Management roles (5, 20)

| Contrast | Treatment comparison |
| --- | --- |
| Management |  |
| IC vs. MC | IC(FS, E, T, CE) vs. MC(FS, E, T, CE) |
| MC vs. IO | MC(E, T, C, CE, CT, ET) vs. IO(E, T, C, CE, CT, ET) |
| IO vs. LO | IO(E, CE) vs. LO(E, CE) |
| IC vs. IO | IC(E, T, CE) vs. IO(E, T, CE) |
| Shade type |  |
| FS vs. shaded  Erythrina vs. FS* | FS(IC, MC) vs. E(IC, MC) + T(IC, MC) + CE(IC, MC)  E(IC, MC) vs. FS(IC, MC) |
| Service vs. timber trees | E(MC, IO) vs. T(MC, IO) + C(MC, IO) + TC(MC, IO) |
| Legume timber vs. non-legume timber | C(MC, IO) vs. T(MC, IO) |

IC: Intensive Conventional, MC: Moderate Conventional, IO: Intensive Organic, LO: Low Organic; FS: Full Sun, E: *Erythrina poepiggiana*, C: *Chloroleucon eurycyclum*; T: *Terminalia Amazonia*; CE:  *Chloroleucon eurycyclum* and *Erythrina poepiggiana*; CT: *Chloroleucon eurycyclum* and *Terminalia Amazonia*; ET: *Erythrina poepiggiana* and *Terminalia Amazonia*;

* Deemded to be the low canopy tree that had a decreased shade cover, Erythrina was relative to full sun.


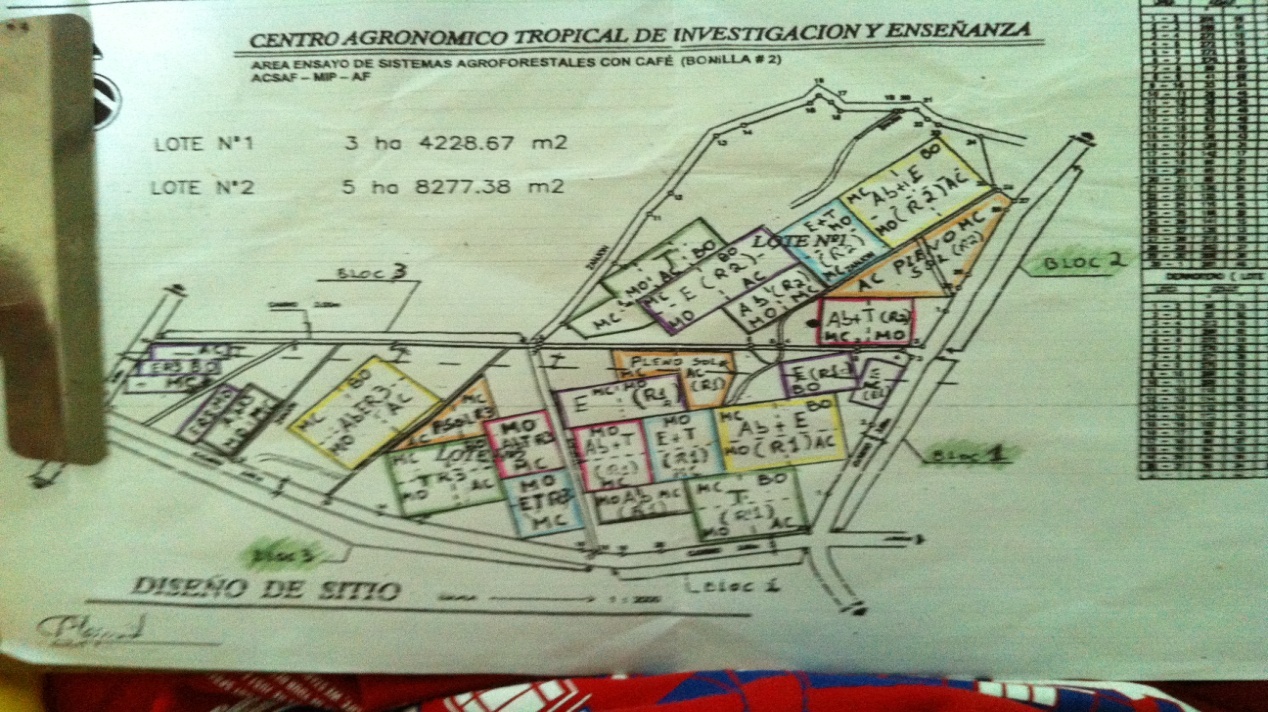


**Figure S1.** Map of three blocks (three replicates) in the field
